# Involvement of hypoxia-inducible factor1-alpha in the protective effect of rivaroxaban against testicular ischemia-reperfusion in rats

**DOI:** 10.1038/s41598-025-10395-2

**Published:** 2025-07-29

**Authors:** Marwan Abdel Baset, Sally A. El Awdan, Marwa S. Khattab, Salma A. El-Marasy

**Affiliations:** 1 Department of Pharmacology, Medical Research and Clinical Studies Institute, National Research Centre, Giza, Egypt; 2 Department of Pathology, Faculty of Veterinary Medicine, Cairo University, Giza, Egypt

**Keywords:** Testicular ischemia-reperfusion, Rivaroxaban, HIF-1α, VEGF, oxidative stress, Apoptosis, Biochemistry, Inflammation

## Abstract

This study investigated the protective effects of Rivaroxaban (RVX) against testicular ischemia-reperfusion (IR) injury in rats with a secondary aim of studying the involvement of hypoxia-inducible factor1-alpha testicular protection against ischemic insults. Twenty-four male rats were divided into four groups: sham control, testicular IR, and two RVX treatment groups (7 and 14 mg/kg) administered for one week prior to IR. Testicular IR led to significant impairment in testicular function, evidenced by an 86.5% reduction in testosterone levels and marked oxidative stress with an 189.3% increase in malondialdehyde (MDA). IR injury also triggered substantial elevations in apoptotic markers (271% increase in Bax (Bcl2-associated X protein) and 65.9% decrease in BCL2 (B-cell lymphoma 2), and inflammatory mediators (285.7% increase in NFκB (nuclear factor-kappa B). Additionally, angiogenic markers showed dramatic increases, with VEGF (vascular endothelial growth factor) and HIF-1 (Hypoxia inducible factor-1) rising by 431.8% and 519%, respectively. RVX treatment demonstrated dose-dependent protective effects, with the 14 mg/kg dose showing superior outcomes compared to 7 mg/kg. The higher dose significantly improved hormonal function (486.8% increase in testosterone), reduced oxidative stress (51.8% reduction in MDA), modulated apoptotic markers (68.3% decrease in BAX, 159.1% increase in BCL2), and normalized angiogenic factors (71.8% reduction in HIF-1). In conclusion, RVX demonstrated significant therapeutic potential in protecting against testicular IR injury, with the 14 mg/kg dose showing optimal protective effects. Reduction in HIF-1α and VEGF protein expression mediated RVX’s anti-oxidant, anti-inflammatory, and anti-apoptotic effect in rats subjected to testicular IR.

**Supplementary Information:**

The online version contains supplementary material available at 10.1038/s41598-025-10395-2.

## Introduction

Testicular ischemia-reperfusion (IR) is one of the most serious conditions, which can lead to infertility if not promptly treated in a timely^1^. Testicular torsion is the most common cause of IR injury. It occurs in 1 in 4,000 males under 25 years of age; the peak incidence is during adolescence^2^. The condition requires urgent medical care as prolonged ischemia followed by reperfusion can trigger a cascade of pathophysiological events leading to irreversible testicular damage^3^.

IR injury pathogenesis in the testis involves several distinct mechanisms. These mechanisms are primarily centered around oxidative stress and inflammatory responses^3,4^. During ischemia, there is a lack of blood flow due to hypoxia and an accumulation of toxic metabolites. Thereafter, reperfusion paradoxically causes further tissue injury through the production of reactive oxygen species (ROS) and pro-inflammatory mediators^4,5^. This oxidative burst sets the apoptotic machinery and disrupts the blood-testis barrier, with serious implications on spermatogenesis and the development of hormonal functions^4^.

Hypoxia-inducible factor-1 (HIF-1) has been shown to play a critical role in the cellular response to oxygen deprivation during testicular IR injury^6–8^. This master regulator of oxygen homeostasis, when activated, initiates the transcription of several genes involved in angiogenesis, like vascular endothelial growth factor (VEGF), and is implicated in the modulation of cellular adaptation to hypoxia^9^. Recent studies have also demonstrated that upregulation of HIF-1 is associated with increased oxidative stress markers and pro-inflammatory cytokines in testicular tissue, so it might also be a therapeutic target to consider in the management of IR injury^10^.

Caspase 3 is an important executioner protease in the apoptotic pathway. It represents a convergence point for both the extrinsic and intrinsic apoptotic signals in testicular IR injury^11^. Its activation is closely related to the oxidative stress response since an increase in ROS level promotes mitochondrial dysfunction. This leads to the release of cytochrome c, leading to enhanced activity of caspase-3^12^. Importantly caspase-3 activation exhibits a bidirectional relationship with the inflammatory mediators, mainly tumor necrosis factor-alpha (TNF-α). Each potentiates the others’ effects and may further establish vicious feedback in IR injury^13,14^. Caspase-3’s relationship to impaired testosterone production suggests its role in Leydig cell dysfunction.

Similarly, its association with increased BAX/BCL2 (Bcl2-associated X protein/ B-cell lymphoma 2),ratio and increased HIF-1α expression identifies its central position in coordinating both death signals and hypoxic responses^15,16^. Understanding these interactions is important for the development of targeted therapeutic strategies that may have the potential to interfere with several pathological pathways all at once.

TNF-α is an important inflammatory mediator in testicular ischemia-reperfusion (IR) injury that orchestrates early inflammatory events and later tissue remodeling^28^. The relationship between TNF-α (tumor necrosis factor-alpha) and oxidative stress represents a feedback loop. This feedback increased the levels of TNF-α and ROS production via mitochondrial disruption. In contrast, augmented oxidative stress, in turn, stimulates TNF-α release^10,17^. Interestingly, TNF-α is significantly correlated with both apoptotic markers and angiogenic factors: it downregulates BCL2 and upregulates BAX and acts synergistically with HIF-1 to increase VEGF production under ischemic conditions^18,19^. New information shows that TNF-α also disrupts Leydig cell function, probably contributing to reduced testosterone levels reported in IR injury. This participation is in both structural and functional testicular dysfunction^20^. These complex interactions of TNF-α with numerous pathways imply that modulating this cytokine could yield synergistic protective benefits in ischemia-reperfusion injury^21^.

Recent evidence suggests that the coagulation cascade plays a central role in IR injury and that thrombosis and microvascular dysfunction significantly contribute to tissue damage^22,23^. A 2022 meta-analysis review highlighted the critical contribution of microvascular occlusion to testicular damage following reperfusion^24^.

This has drawn increasing interest in the potential therapeutic use of anti-coagulants in the treatment of IR injury^25^. Rivaroxaban (RVX), a direct inhibitor of factor Xa, has shown promising effects in various IR injury models of cardiac and cerebral tissues^26,27^. Its anti-coagulant properties, with probably anti-inflammatory and anti-oxidant properties, make it a very promising candidate in the management of testicular IR injury. Recent research revealed that factor Xa inhibition significantly reduces inflammation and oxidative stress in multiple organ systems following IR injury^28^. RVX’s anti-coagulant properties, combined with its potential anti-inflammatory and anti-oxidant effects, make it a particularly promising candidate for testicular IR injury management.

RVX offers several advantages over other potential therapeutic agents for testicular IR injury. First, it has excellent oral bioavailability and established pharmacokinetic properties^29^. Second, it specifically targets factor Xa, a crucial component in the thrombosis that follows reperfusion. Third, emerging evidence suggests RVX possesses pleiotropic effects beyond anti-coagulation that may directly address the multiple pathways involved in IR injury pathogenesis^30,31^.

Currently, surgical detorsion remains the standard treatment for testicular torsion. However, this approach alone cannot prevent subsequent reperfusion injury^32^. Although several therapeutic agents have been tried to evaluate their protective effects against testicular IR injury. Unfortunately, most of them have shown limited efficacy or face practical limitations in clinical settings^4^. Thus, effective pharmacological interventions are desired for administration in a preventive manner or in the early stages of reperfusion. These would ideally be administered preventively or during the early stages of reperfusion to address this significant clinical challenge^5^.

Although RVX has been established to play a protective role in preventing thrombotic events, its potential protective effects against testicular IR injury have not been fully elucidated. The present study aimed to investigate three specific objectives: (1) evaluate the protective effects of two different RVX doses (7 and 14 mg/kg) against testicular IR injury in rats; (2) determine the involvement of HIF-1α in these protective effects; and (3) characterize the relationships between HIF-1α, oxidative stress parameters, inflammatory mediators, and apoptotic markers following RVX treatment. Understanding these dose-dependent effects could provide valuable insights for developing more effective therapeutic strategies for testicular IR injury in clinical settings.

### Animals

Twenty-four mature male Wistar rats weighing 180–200 g were purchased from the National Research Centre’s Animal House Facility after the Ethical Committee for Medical Research, Egypt, approved the animal research protocol (permission No. 01460124). Animals were housed in standard cages under pathogen-free circumstances, with controlled room temperature and typical dark-light cycles.

Rats were fed regular diets and given unlimited access to water. Before beginning the experimental regimen, the animals were allowed a week to adjust to their surroundings.

### Drugs and chemicals

RVX was purchased from Inspire Pharmaceutical Company in Cairo, Egypt. All other compounds were of analytical grade and bought from commercial suppliers.

### Induction of testicular ischemia

Testicular ischemia in rats was induced by the torsion and detorsion (T/D) of the left testis for 2 h, as described before^33^. Before surgery, xylazine hydrochloride (10 mg/kg) and ketamine (100 mg/kg) were injected intraperitoneally (ip) to create general anesthesia^34^. To keep the rats’ core body temperature at 37 C, they were put on a homeothermic table.

All procedures were carried out in a sterile environment. Following anesthesia, the left testis and its spermatic cord were revealed following a vertical paramedian incision of the scrotum. The left testicle was rotated clockwise with its cord 720 to produce a unilateral testicular torsion. 6/0 propylene suture was used to secure the testicle to the inside of the scrotum. After 120 min of torsion, the left testis was deformed back into its original normal position and reinserted into the scrotum to accomplish reperfusion.

## Experimental design

Twenty-four adult male rats weighing 180–200 g will be randomly allocated into 4 groups (6 rats/group).

In group 1, the sham group (Rats were treated with the vehicle (distilled water). They underwent the same surgical procedure, with the exception of T/D of the left testis. Group 2 testicular IR group. Group 3 and 4 were administered RVX orally in doses of 7 and 14 mg/kg, respectively for 1 week prior to IR^35^.

Following reperfusion, blood samples were drawn from the rats’ retroorbital venous plexus using a needle and centrifuged at 3000 rpm for 10 min in nonheparinized tubes, to separate the serum^36^. The supernatant serum was collected and stored at − 80 °C for further investigation. Then, rats were euthanized by decapitation under thiopental sodium (200 mg/kg, i.p.) anesthesia^37^; the left testicles were taken out and split in half. For additional biochemical study, one portion was kept at 80 °C, while the other was kept for histopathological and immunohistochemical analysis.

Following the manufacturer’s instructions, an ELISA kit (Cusabio, Wu Han, China) was utilized for the quantitative protein detection of testosterone (Catalog no: CSB-E05100r).

### Preparation of tissue homogenates

The left testes were homogenized (MPW-120; Medical Instruments) in 10% (w/v) ice-cold phosphate buffer. The homogenate was then centrifuged for 5 min in a cooling centrifuge (2 k15; Sigma/Laborzentrifugen). The resultant supernatants were collected and kept at 80 degrees Celsius for further analysis.

### Assessment of oxidative stress markers in testicular homogenate

The assay kits obtained from Biodiagnostic Company (Cairo, Egypt) were used to measure the levels of glutathione peroxidase (GPX)^38^ (catalog no. GP 2524) and malondialdehyde (MDA)^39^ (catalog no. MD 2529),in compliance with the procedures specified in the manufacturer’s instructions.

### Estimation of inflammatory biomarkers in testicular homogenate

Using the My BioSource (San Diego, USA) ELISA kit with catalog number MBS2505513, nuclear factor kappaB-p65 (NF-ĸb p65) was measured in the left testis homogenate in accordance with the manufacturer’s instructions.

### Determination of apoptotic biomarkers

Rat ELISA kits, with catalog numbers CSB-EL002573RA and abx155248 respectively, from Cusabio (Wu Han, China) and Abbexa Ltd (Cambridge, UK) were used to measure Bax and BCL2, respectively.

### Western blot

The protein extracts of HIF-1alpha and VEGF from testis tissues were measured using the Bradford method. We separated equal amounts of the extracted proteins by “SDS-PAGE” (10% acrylamide gel), followed by their transfer to polyvinylidene difluoride (PVDF) membranes (BioRad, CA, USA). After transfer, we washed the membranes using Phosphate buffered saline and blocked them with 5% skimmed milk powder in PBS for one hour at room temperature. After that, they were incubated at 4◦C with antibodies provided by (Thermo Scientific, Rockford, Illinois, USA) for the whole night. Following washing, “Peroxidase-labeled secondary antibodies” were added, and we incubated the membranes at 37◦C for one h. The “ChemiDocTM imaging system” analyzed band intensity with Image LabTM software version 5.1 (Bio-Rad Laboratories Inc., Hercules, CA, USA).

### Histopathological examination of the testis

Testicular tissues were placed in 10% neutral buffered formalin for processing by ethanol and xylene, embedding in wax, sectioning (3–4 μm), and staining by hematoxylin and eosin stain^40^. A digital camera (Olympus XC30, Tokyo, Japan) connected to light microscope was used to capture images.

Cosentino scoring was carried out on the testes in which Grade 1 indicated no testicular lesion, Grade 2 indicated separation of germ cells in well-arranged seminiferous tubules, Grade 3 indicated disorganized and desquamated germ cells with undulating basement membrane of seminiferous tubules, and Grade 4 indicated coagulative necrosis in germ cells present in damaged seminiferous tubules^41^. Johnsen’s spermatogenesis score was performed in 10 seminiferous tubules according to previous research^42^. Score 1 indicates the absence of spermatogonia, score 2 indicates germinal aplasia, score 3–7 indicates maturation arrest, score 8 indicates hypospermatogenesis, score 9 indicates mild alteration and score 10 indicates full spermatogenesis and perfect tubules.

### Immunohistochemistry

After deparaffinization and rehydration of paraffin-embedded tissue sections, slides were incubated with citrate buffer PH 6 for antigen retrieval. Anti-TNF-α (Santa Cruz, USA) and Anti-caspase-3 (Abexxa, UK) were incubated with tissue overnight. Blocking of endogenous peroxidase was then performed followed by the application of secondary antibodies provided by the universal kit (Bio SB, USA). Counterstain with Hematoxylin was applied. Captured images from light microscope were analyzed by Image J software to measure the brown positive area percentage in 3 micrographs at 200 X magnification power for each rat^43,44^.

### Statistical analysis

The variability from the results is expressed as the mean ± standard error of the mean (SEM). The Brown-Forsythe Test tested the standard deviation of the data, while the normality test was applied by the Shapiro-Wilk test. The data satisfying these conditions were evaluated using one-way ANOVA and afterward ranked using Tukey-Kramer post hoc multiple comparisons. The Johnsen and Cosentino scores in the pathology, did not fit the above condition, and only their median value obtained by the following method was subjected to the Kruskal-Wallis test and Dunn’s multiple comparisons.

### Ethics declaration

All methods were performed in accordance with the relevant guidelines and regulations of the Ethical Committee for Medical Research of the National Research Centre in Egypt with approval number 01460124. All experimental procedures were conducted in accordance with ARRIVE guidelines.

## Results

### Impact of Rivaroxaban on testicular hormonal function

**Fig. 1:**
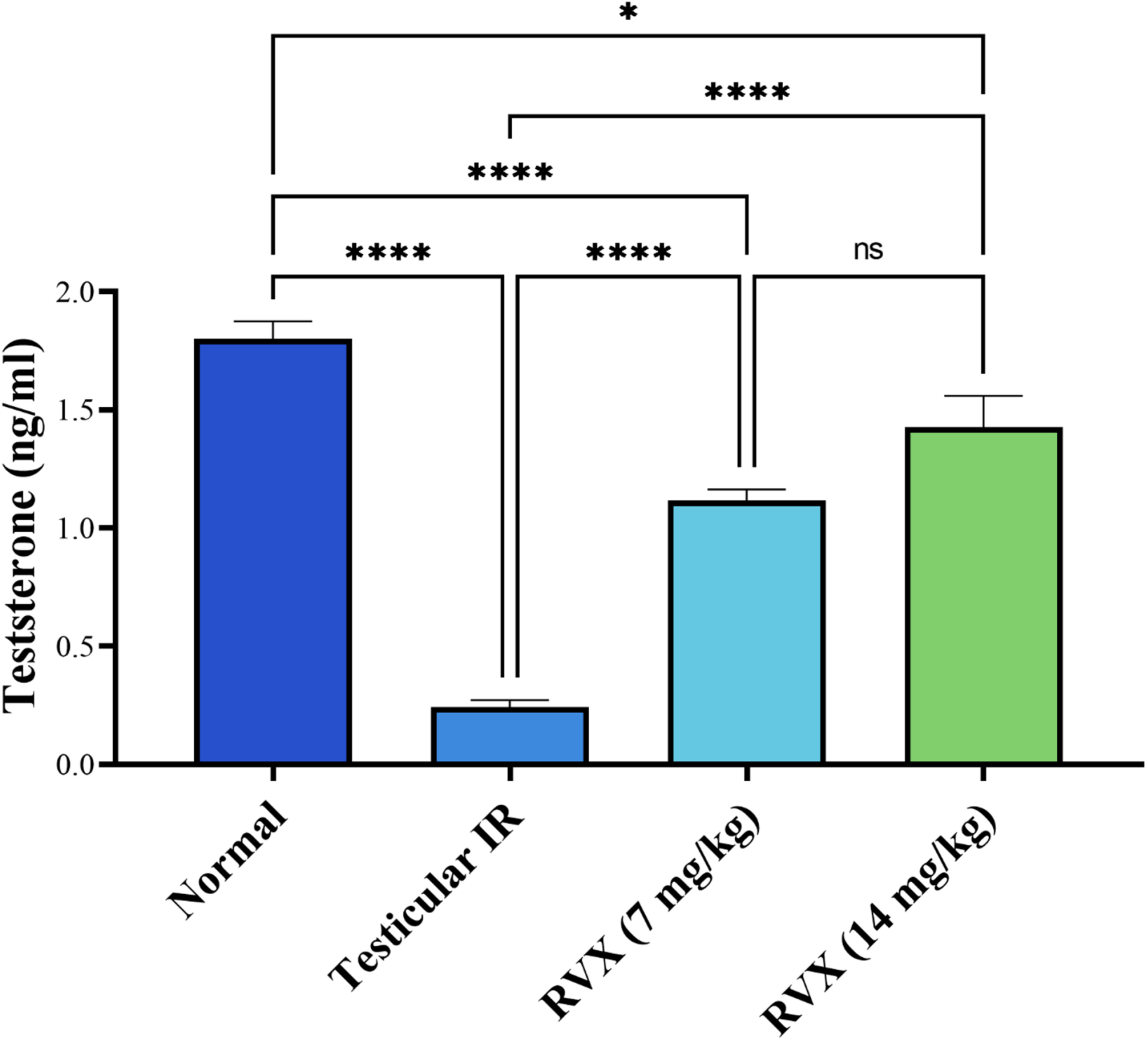
Effects of rivaroxaban on serum testosterone against testicular ischemia-reperfusion injury in rats. Each bar represents the mean ± SEM of 6 rats: * vs. corresponding pairwise at *p*< 0.05, ** vs. corresponding bar at *p*< 0.01, *** vs. corresponding bar at *p*< 0.001, **** vs. corresponding bar at *p*< 0.0001.

As depicted in Fig. 1, testicular IR induced a severe reduction in testosterone levels, showing an 86.5% decrease compared to the normal group. Treatment with RVX showed dose-dependent improvement, with the 7 mg/kg dose increasing testosterone by 358.8% and the 14 mg/kg dose increasing it by 486.8% compared to the IR group. While both doses significantly improved testosterone levels, neither fully restored it to normal values, with the 7 mg/kg and 14 mg/kg doses remaining 38.1% and 20.8% below normal levels, respectively. The higher dose (14 mg/kg) improved significantly better than the lower dose.

### Impact of Rivaroxaban on oxidative stress markers in testicular tissue

**Fig. 2:**
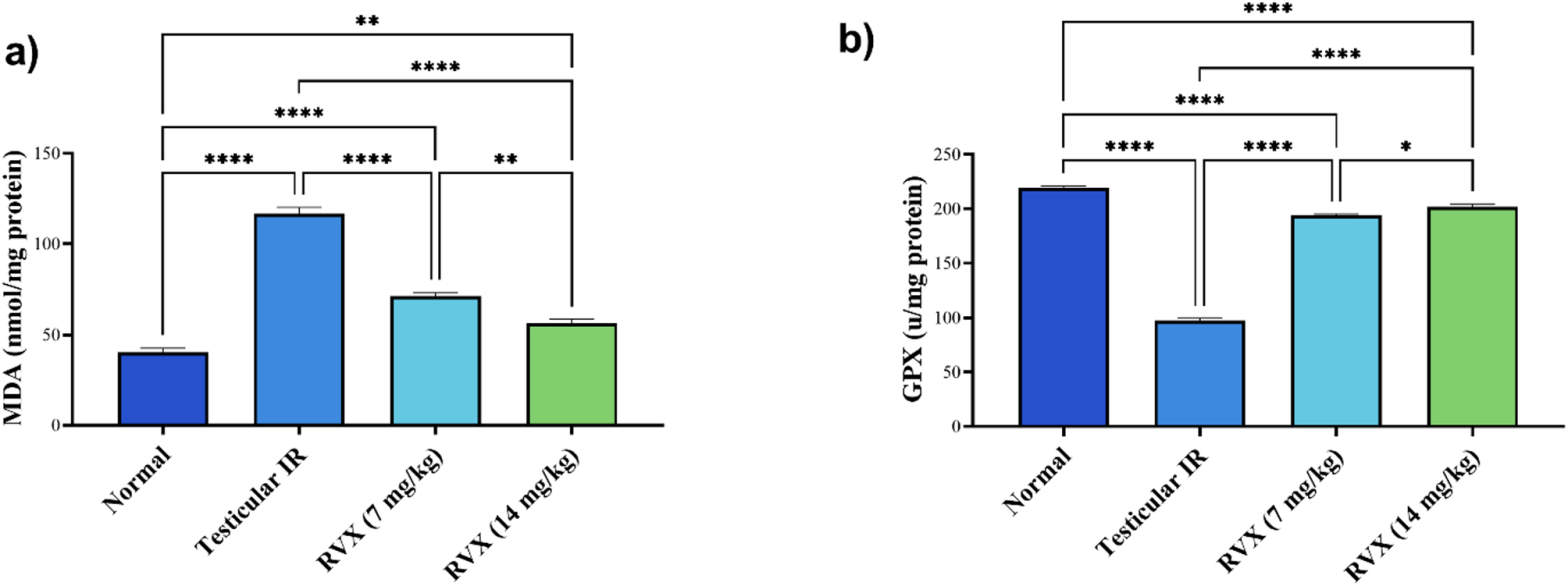
Effects of rivaroxaban on testicular (**a**) MDA and (**b**) GPX content against testicular ischemia-reperfusion injury in rats. Each bar represents the mean ± SEM of 6 rats: * vs. corresponding pairwise at *p*< 0.05, ** vs. corresponding bar at *p*< 0.01, *** vs. corresponding bar at *p*< 0.001, **** vs. corresponding bar at *p*< 0.0001.

As shown in Fig. 2a, testicular IR caused significant oxidative stress, evidenced by an 189.3% increase in MDA levels compared to the normal group. Treatment with RVX demonstrated significant anti-oxidant effects, with the 7 mg/kg dose reducing MDA by 39.1% and the 14 mg/kg dose reducing it by 51.8% compared to the IR group. However, both doses still showed elevated oxidative stress compared to normal levels, with MDA remaining 76.3% and 39.5% above normal for 7 mg/kg and 14 mg/kg doses, respectively. The 14 mg/kg dose showed significantly better anti-oxidant effects than the 7 mg/kg dose.

As depicted in Fig. 2b, Testicular IR resulted in severe impairment of anti-oxidant defense mechanisms, as evidenced by a 55.5% reduction in GPX levels compared to the normal group. Treatment with RVX resulted in remarkable improvement in the restoration of anti-oxidant capacity, with the 7 mg/kg dose increasing GPX by 98.3% and the 14 mg/kg dose elevating it by 106.7% compared to the IR group. While the two doses significantly improved GPX levels, they did not quite reach normal values; the 7 mg/kg and 14 mg/kg doses remained 11.8% and 8.1% below normal levels, respectively. The higher dose thus showed a marginally better restoration of anti-oxidants and hence better protection against oxidative stress.

### Impact of Rivaroxaban on apoptotic and inflammatory markers

**Fig. 3:**
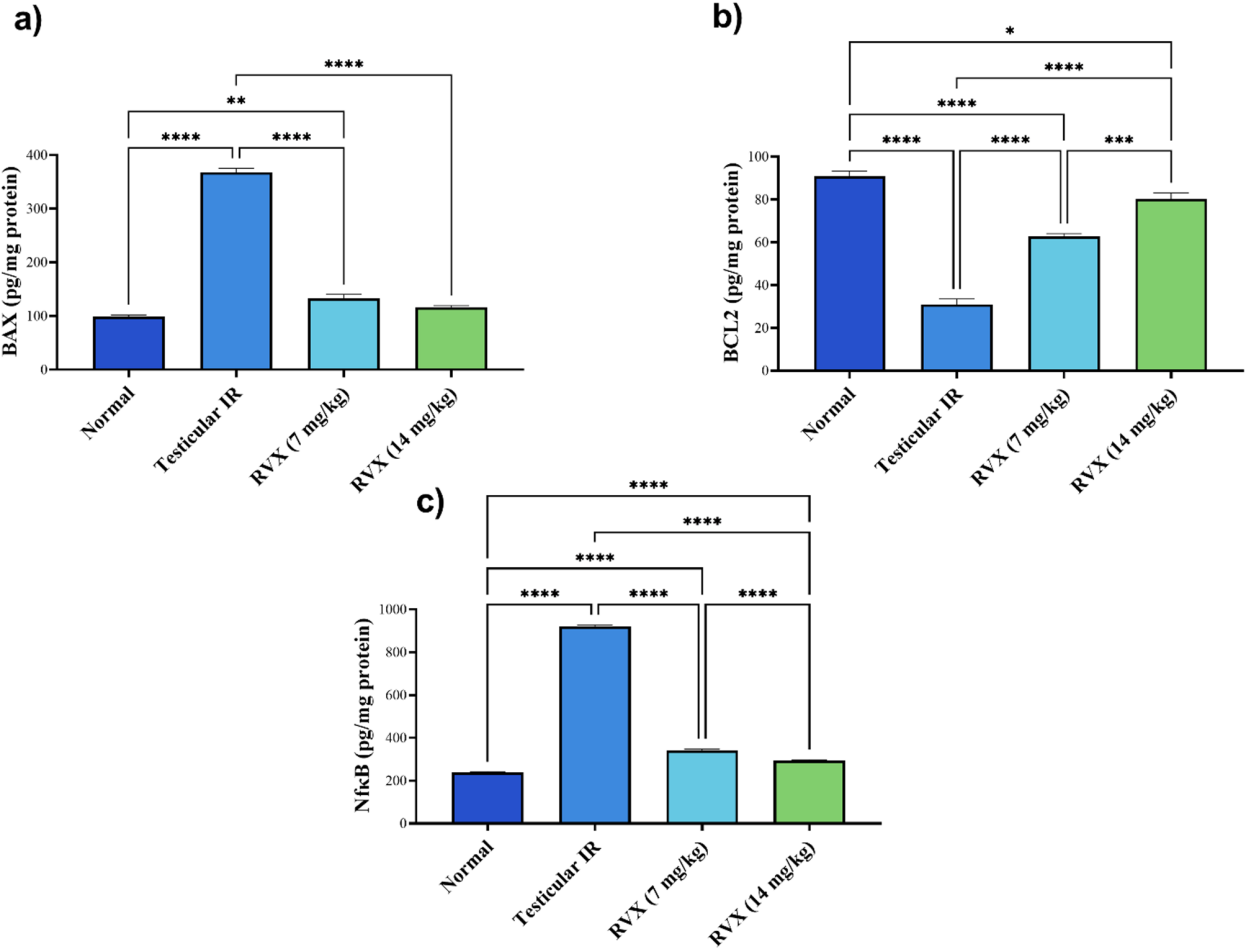
Effects of rivaroxaban on testicular (**a**) BAX and (**b**) BCL2 (**c**) NF-ĸb p65 content against testicular ischemia-reperfusion injury in rats. E Each bar represents the mean ± SEM of 6 rats: * vs. corresponding pairwise at *p*< 0.05, ** vs. corresponding bar at *p*< 0.01, *** vs. corresponding bar at *p*< 0.001, **** vs. corresponding bar at *p*< 0.0001.

Figure 3 reveals that testicular IR dramatically increased apoptotic markers, with BAX increasing by 271% and NFκB increasing by 285.7% while BCL2 decreased by 65.9% compared to normal controls. RVX treatment at 7 mg/kg reduced BAX by 63.9% and NFκB by 62.9%, while increasing BCL2 by 102.8% compared to the IR group. The 14 mg/kg dose showed even better effects, reducing BAX by 68.3% and NFκB by 68.1%, while increasing BCL2 by 159.1% compared to IR. Neither dose completely normalized these markers, but the 14 mg/kg dose showed significantly better anti-apoptotic and anti-inflammatory effects than the 7 mg/kg dose.

Analysis of the BAX/BCL2 ratio, a critical indicator of apoptotic tendency, revealed dramatic alterations across the experimental groups. The ratio increased substantially from 1.09 in the normal group to 11.88 in the IR group, representing a striking 989.2% elevation and indicating a severe shift toward proapoptotic signalling. RVX treatment demonstrated marked effectiveness in restoring this balance, with the 7 mg/kg dose reducing the ratio by 82.2% to 2.12. In comparison the 14 mg/kg dose showed superior effectiveness, reducing the ratio by 87.8% to 1.45, bringing it closer to the normal baseline. The dose-dependent improvement in BAX/BCL2 ratio suggests RVX’s potent anti-apoptotic effects, with the higher dose providing more effective protection against IR-induced apoptotic signalling.

### Impact of Rivaroxaban on VEGF expression in testicular tissue

**Fig. 4:**
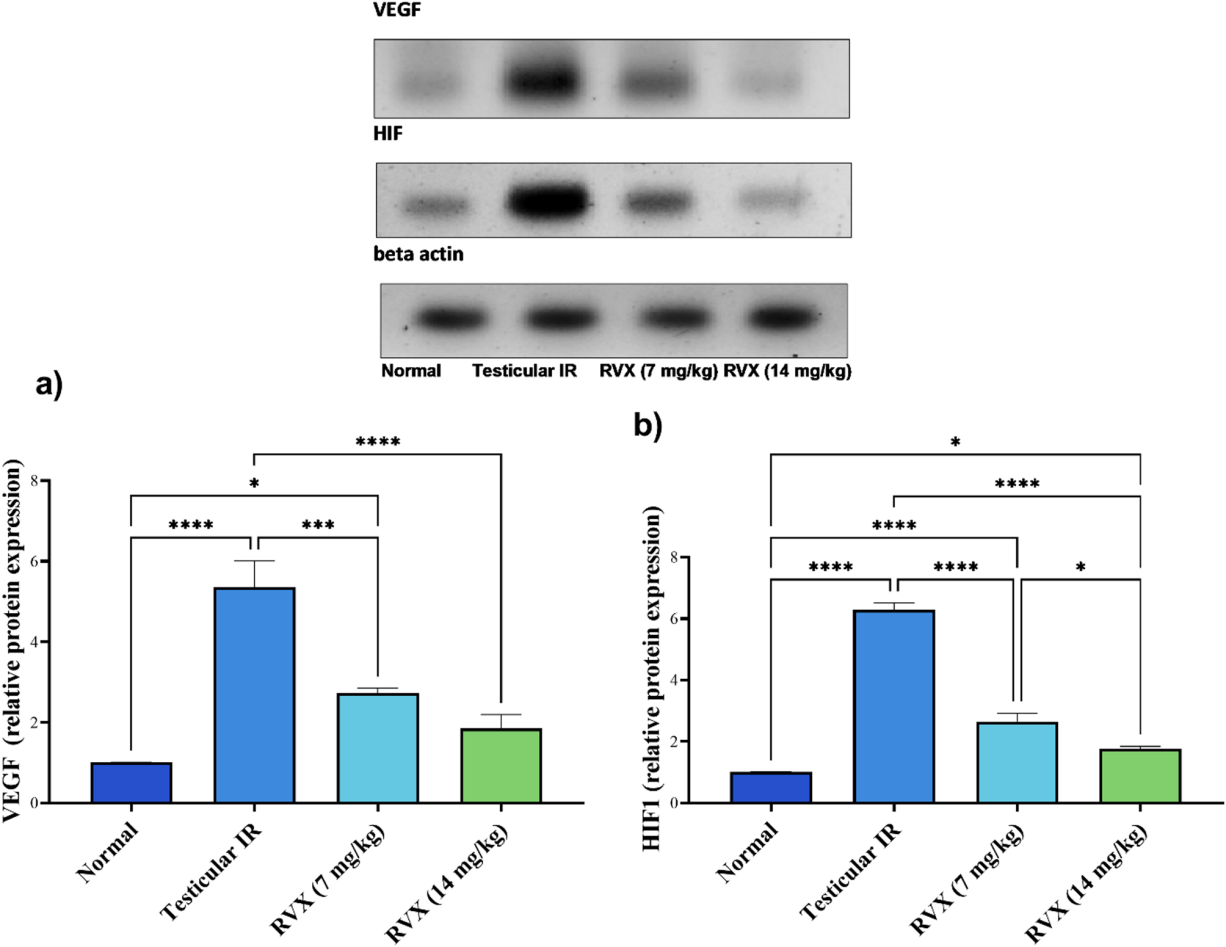
Effects of rivaroxaban on testicular (**a**) VEGF and (**b**) HIF1 content against testicular ischemia-reperfusion injury in rats. Each bar represents the mean ± SEM of 6 rats: * vs. corresponding pairwise at *p*< 0.05, ** vs. corresponding bar at *p*< 0.01, *** vs. corresponding bar at *p*< 0.001, **** vs. corresponding bar at *p*< 0.0001.

VEGF expression in Fig. 4a was markedly elevated in the IR group, showing a 431.8% increase compared to normal controls. RVX treatment showed dose-dependent effects in normalizing VEGF expression, with the 7 mg/kg dose reducing it by 49% and the 14 mg/kg dose reducing it by 65.4% compared to the IR group. However, both doses maintained elevated VEGF levels compared to normal (171.3% and 83.7% above normal for 7 and 14 mg/kg, respectively), with the 14 mg/kg dose showing significantly better normalization than the 7 mg/kg dose.

### Impact of Rivaroxaban on HIF-1 expression analysis

HIF-1 expression in Fig. 4b increased dramatically by 519% in the IR group compared to normal controls. RVX treatment showed significant dose-dependent effects, with the 7 mg/kg dose reducing HIF-1 expression by 57.9% and the 14 mg/kg dose reducing it by 71.8% compared to the IR group. While both doses of RVX significantly improved HIF-1 expression, neither fully normalized it (160.6% and 74.3% above normal for 7 and 14 mg/kg, respectively). The 14 mg/kg dose demonstrated significantly better regulation of HIF-1 expression compared to the 7 mg/kg dose.

### Histopathological findings

**Fig. 5:**
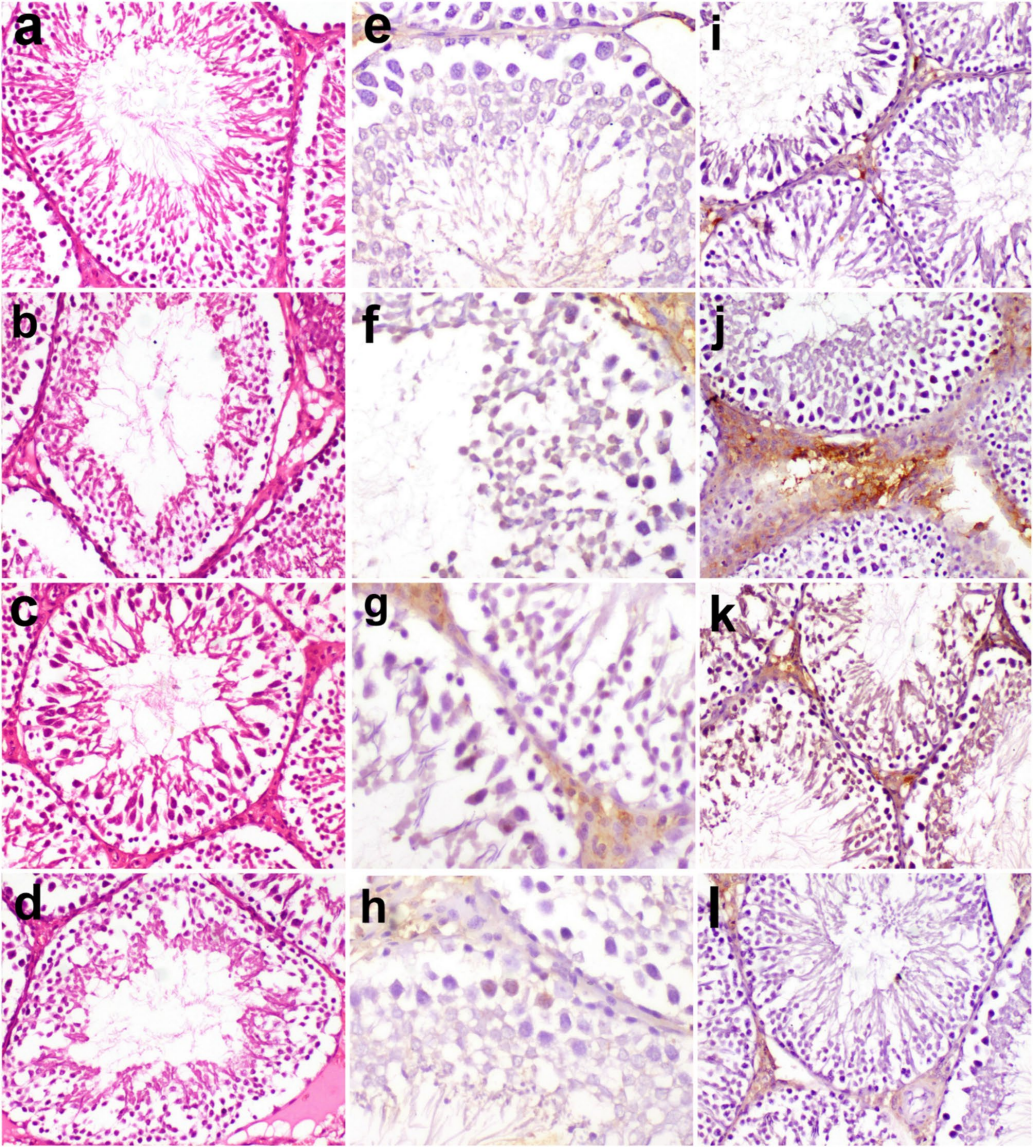
Effects of rivaroxaban on histopathology and immunohistochemistry of caspase-3 and TNF-alpha in testicular ischemia-reperfusion injury in rats. (**a**) Normal histological structure of seminiferous tubules with full spermatogenesis in normal group. (**b**) Impaired spermatogenesis, distorted germinal epithelium and few early spermatids in IR group. (**c**) The spermatogenesis was slightly improved and the germinal epithelium lining seminiferous tubules showed moderate alteration in RVX (7 mg/kg). (**d**) The spermatogenesis was slightly impaired, and seminiferous tubules had many late spermatids in RVX (14 mg/kg). hematoxylin and eosin stain (X200). (**e**) Caspase-3 immunostaining was weak in normal group, (**f**) severe in Leydig cells, many spermatocytes and few spermatogonia in seminiferous tubules of IR group, (**g**) moderate in spermatocytes and Leydig cells of RVX (7 mg/kg). (**h**) few positive spermatocytes and Leydig cells in RVX (14 mg/kg). (Immunoperoxidase X400). (**i**) TNF-α was expressed weakly in Leydig cells and interstitial tissue of Normal group, (**j**) severe in IR group, (**k**) moderate in RVX (7 mg/kg) group, (**l**) and mild in RVX (14 mg/kg) group. (Immunoperoxidase X200).

Testicular microscopy in the normal group revealed a normal histological structure of seminiferous tubules with full spermatogenesis (Fig. 5a). In the testicular IR group, the seminiferous tubules showed impaired spermatogenesis, distorted germinal epithelium and few early spermatids (Fig. 5b). In RVX (7 mg/kg) group, the spermatogenesis was slightly improved and the germinal epithelium lining seminiferous tubules showed moderate alteration compared to testicular IR group (Fig. 5c). In RVX (14 mg/kg), the spermatogenesis was slightly impaired, and seminiferous tubules had many late spermatids (Fig. 5d).

### Immunohistochemical findings

Caspase-3 immunostaining was weak in the normal group (Fig. 5e). However, it was observed in Leydig cells, many spermatocytes and few spermatogonia in seminiferous tubules of the IR group (Fig. 5f). In RVX (7 mg/kg) group, there was moderate staining of spermatocytes and Leydig cells (Fig. 5g). In RVX (14 mg/kg), there were few positive spermatocytes and Leydig cells (Fig. 5h).

TNF-α was expressed mostly in Leydig cells and interstitial tissue. The expression was weak in the normal group (Fig. 5i), severe in the testicular IR group (Fig. 5j), moderate in the RVX (7 mg/kg) group (Fig. 5k) and mild in RVX (14 mg/kg) group (Fig. 5l).

**Fig. 6:**
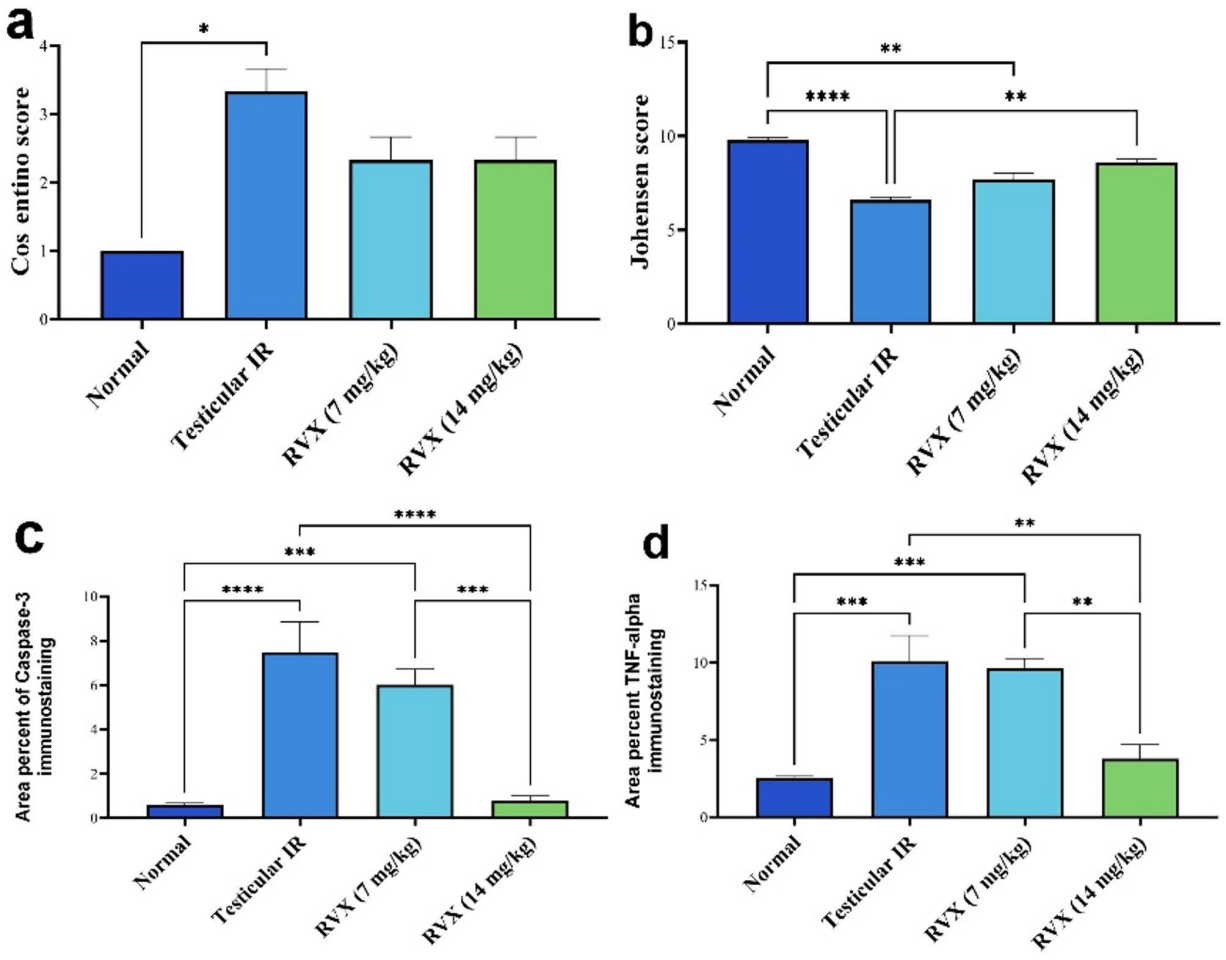
Effects of rivaroxaban on (**a**) Cosentino’s score, (**b**) Johnsen’s score, (**c**) Testicular caspase-3 Expression (**d**) Testicular TNF-alpha against testicular ischemia-reperfusion injury in rats. Each bar represents the mean ± SEM of 6 rats: * vs. corresponding pairwise at *p*< 0.05, ** vs. corresponding bar at *p*< 0.01, *** vs. corresponding bar at *p*< 0.001, **** vs. corresponding bar at *p*< 0.0001.

According to cosentino’s and Johnsen’s scores, spermatogenesis was remarkably impaired in the testicular IR group whereas it was improved in RVX (14 mg/kg) (Fig. 6a, b).

Caspase-3 and TNF-α expression were significantly elevated in testicular IR. However, caspase-3 and TNF-α protein expression was decreased in RVX (14 mg/kg) (Fig. 6c, d).

## Discussion

To the best of the author’s knowledge, this is the first study to reveal the preventive benefits of RVX against testicular ischemia-reperfusion (IR) damage in rats. Our results indicated that testicular IR caused considerable functional and structural impairment. This was witnesses by reduction testosterone levels, elevation of oxidative stress indicators, intensification of inflammatory responses, and activation of apoptotic pathways. The administration of RVX demonstrated dose-dependent protective effects, with the higher dosage (14 mg/kg) offering more protection than the lower dosage (7 mg/kg).

Testicular IR results in testicular dysfunction and male infertility. Testicular torsion causes venous congestion, arterial blockage, and ischemic and necrotic changes, which impede testicular perfusion^4^. Testicular detorsion contributes to reperfusion of the ischemic tissue triggering morphological and biochemical alterations resulting in the evolution of ROS, inflammation^45^.

A significant discovery was the role of hypoxia-inducible factor 1-alpha (HIF-1α) in the pathophysiology of testicular IR damage. HIF-1α levels surged significantly (519%) after IR damage. The RVX therapy markedly decreased HIF-1α levels, with the 14 mg/kg dosage exhibiting the most substantial impact (71.8% reduction). This indicates that the regulation of HIF-1α is a significant mechanism behind the protective effects of RVX. Additionally, in this study, RVX elevated testosterone levels, reduced protein expressions of VEGF, and thereby reduced hypoxia and promoted spermatogenesis. In accordance, RVX elevated testosterone levels in cisplatin-induced models of testicular damage^35^.

Our results support the hypothesis that HIF-1α serves as a central mediator in testicular IR injury pathogenesis. Under normal conditions, following hydroxylation and ubiquitination, HIF-1α is degraded by the ubiquitin-dependent proteasome 26 S pathway. During ischemia, HIF-1α accumulates due to reduced oxygen-dependent degradation. Upon reperfusion, the accumulated HIF-1α initiates transcription of various genes, including VEGF^46–48^. It has been reported that VEGF combats spermatogonia proliferation and, thereby, spermatogenesis^49^. While HIF-1α activation represents an adaptive response to hypoxia, sustained or excessive activation can become maladaptive^50^. **Palladino**,** Fasano**^48^ demonstrated that prolonged HIF-1α activation contributes to inflammatory responses in testicular tissue. Additionally, prior study showed a direct correlation between elevated HIF-1α levels and increased apoptotic markers in testicular injury^51^.

In accordance, in this study, hypoxia-induced reduction in testosterone level and thereby impaired spermatogenesis; moreover, by upregulating VEGF, hypoxia inhibits spermatogenesis and decreases sperm motility and count^49^. According to our results, the therapy with RVX resulted in a considerable reduction in the levels of HIF-1α. With the larger dosage (14 mg/kg), this effect was more noticeable and had a significant impact. Based on this information, it seems that RVX may exert its protective effects in part via modulating the HIF-1α pathway. Similarly, Abdelzaher et al. (2020) found that dipeptidyl peptidase-4 inhibitors protect against testicular IR damage by regulating HIF-1α^46^. Our work provides more evidence that RVX has comparable effects on HIF-1α in testicular tissue, hence extending the results of previous research.

In this study, RVX reduced testicular MDA and elevated GPx content, revealing that its anti-oxidant effect mediated its protection against testicular IR. The current finding is in harmony with prior studies where RVX possessed an anti-oxidant effect in cisplatin-induced testicular damage^35^, renal injury induced by sunitinib^52^, and sunitinib‑induced cardiotoxicity^53^.Oxidative stress results from the imbalance between the formation of ROS and anti-oxidants; under hypoxic conditions associated with testicular IR, excessive ROS results in oxidative stress^54^. Testes are highly prone to oxidative stress; under normal conditions, anti-oxidants play a key role in maintaining fertility. MDA is an end-product of lipid peroxidation and determines the extent of membrane lipid peroxidation. GPx, an endogenous anti-oxidant enzyme, represents an initiative line of defense against oxidative damage resulting from testicular IR^55^. Therefore, MDA was markedly elevated in testicular IR whereas GPx was declined. It has been demonstrated that hypoxia induces the generation of ROS and oxidative stress^49^, thereby RVX may have exerted its anti-oxidant effect via mitigation of hypoxia and reduction of HIF1-α.

RVX reduced TNF-α protein expression, indicating that NF-ĸB p65 was not translocated into the nucleus, implicating that RVX protected against testicular IR via its anti-inflammatory effect. In line, with this finding RVX exerted anti-inflammatory activity against cisplatin-induced testicular damage^35^ and chronic unpredicted mild stress-induced depression^56^.

ROS production triggers inflammation during testicular IR^57^. This implies that RVX’s anti-oxidant effect mediated its anti-inflammatory activity. Previous studies reported that HIF1-α and VEGF trigger inflammatory cytokines during I/R^58,59^, therefore it can be deduced that RVX exerted its anti-inflammatory effect via lowering testicular protein expression of HIF1-α and VEGF.

In this study, RVX elevated the anti-apoptotic protein BCL2, reduced the apoptotic BAX, reduced the BAX/BCL2 ratio and thereby reduced caspase 3, the end product of apoptosis. Consistent with this finding, RVX combated apoptosis in the myocardial ischemia rat model^60^. The significant elevation in apoptotic markers observed in our study aligns with the complex interplay between inflammatory mediators and the apoptotic cascade in testicular IR injury. TNF-α elevation, as demonstrated in our results, likely serves as an upstream initiator of caspase-3 activation, consistent with previous findings^61^ by showing strong positive correlations between TNF-α levels and caspase-3 activity in testicular tissue following cisplatin-induced testicular damage. The parallel increases in oxidative stress markers (MDA) and inflammatory mediators (NFκB) observed in our study may create a self-amplifying cycle that sustains caspase-3 activation, as suggested by Almarzouq &Al-Maghrebi^62^, Arena, Iacona^63^. This is further supported by Musaogullari, Mandato^64^, who demonstrated that oxidative stress-induced mitochondrial dysfunction enhances caspase-3 activation through the intrinsic apoptotic pathway. The significant improvement in the BAX/BCL2 ratio following RVX treatment (87.8% reduction with 14 mg/kg dose) suggests that RVX may interrupt this cycle by modulating both inflammatory and apoptotic pathways. This dual action has been previously reported by^60^, who showed that anti-coagulant therapy can reduce both TNF-α production and caspase-3 activation in various models of IR injury. Furthermore, the dose-dependent reduction in HIF-1 expression observed with RVX treatment may contribute to decreased caspase-3 activation, as has been demonstrated that sustained HIF-1 expression can sensitize cells to TNF-α-induced apoptosis through enhanced caspase-3 activation^65^. These findings collectively suggest that the protective effects of RVX against testicular IR injury may be partially mediated through the modulation of the TNF-α/Caspase-3 axis, offering a potential therapeutic target for future interventions.

By controlling the permeability of the outer membrane of the mitochondria, Bcl-2 proteins control apoptosis. Increased mitochondrial membrane permeability results from decreased Bcl-2 and increased Bax levels. This results in the activation of caspase-3; which triggers intrinsic apoptosis. Bax enhances the mitochondrial release of cytochrome C, which triggers apoptosis while BCL2 inhibits its release and combat apoptosis.

It has been reported that reduction of HIF1-α reduced germ cell apoptosis and enhanced spermatogenesis^47^. HIF1-α promotes apoptotic cell death by regulating inflammatory response. Moreover, VEGF exhibited proapoptotic properties during IR^58^. Therefore, it can be deduced that RVX reduced the expression of HIF1-α and VEGF, which was associated with a reduction in inflammation and consequently apoptosis.

To sum up, RVX exerted protection against testicular IR by reducing oxidative stress, inflammation, and apoptosis which was mediated by a reduction in the expression of VEGF and HIF1-α. More studies are warranted to deduce another molecular mechanism that will remain unexplored in the current study.

## Study limitations and future directions

It is important to recognize that our research has a few shortcomings. We tested two different dosages of RVX. To establish a comprehensive dose-response relationship, it is recommended that a wider range of dosages be investigated further. Second, the immediate consequences that followed an IR injury were the primary focus of our work. To further understand the long-term effects, especially in relation to fertility, more research is required. There will be an increase in the value of the acquired results provided by the addition of bigger animal cohorts of the appropriate sample size.

The use of animal models is a good starting point, but the need for further translation steps is crucial to confirm the obtained finding. Therefore, the obtained results are recommended to be replicated in clinical settings. Furthermore, while we have proven the effects of RVX on several pathways, the specific molecular processes that connect the inhibition of factor Xa to the regulation of HIF-1α are still not well known.

## Conclusion

**Fig. 7:**
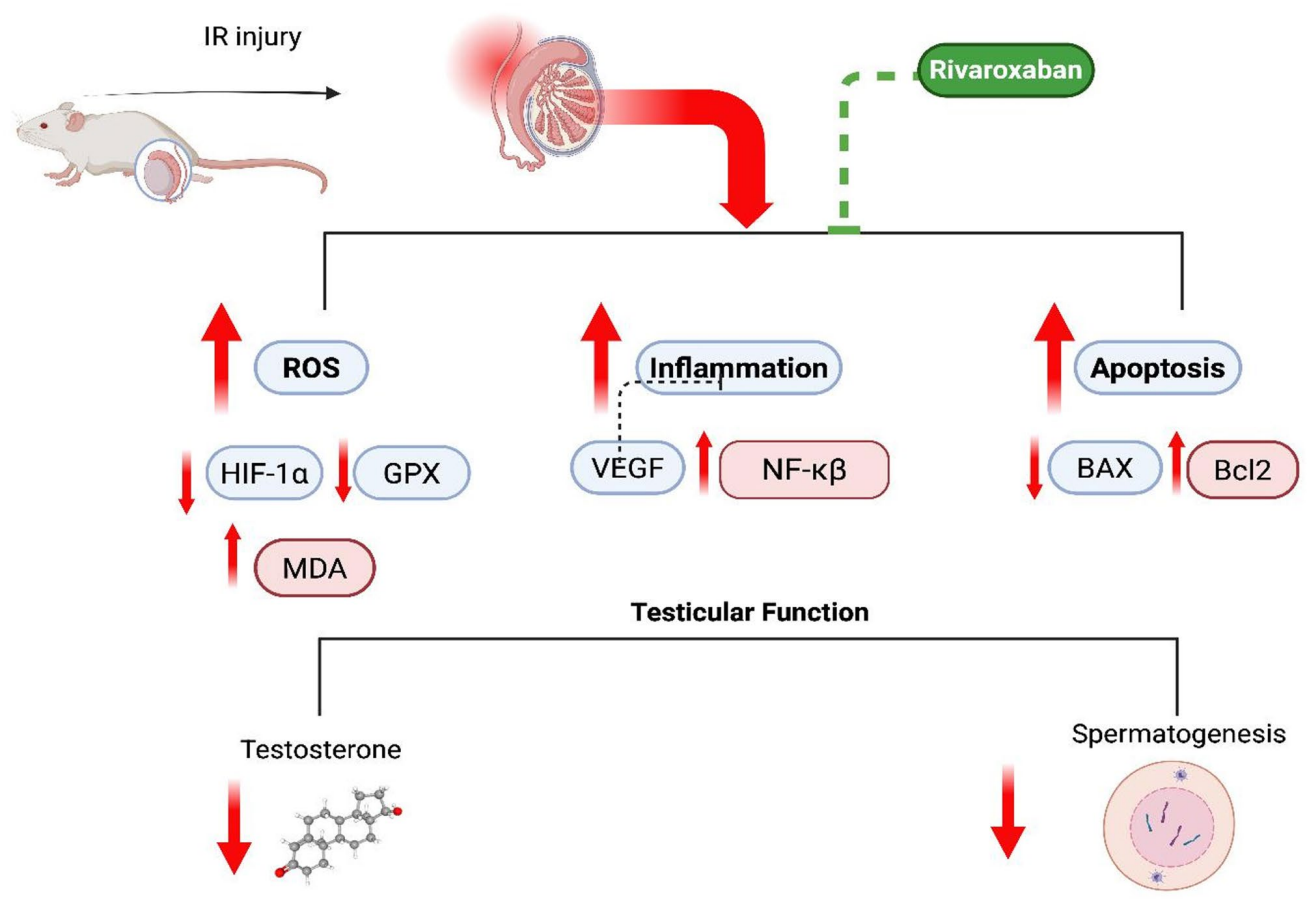
Summary on the involvement of hypoxia-inducible factor1-alpha in the protective effect of Rivaroxaban against testicular ischemia-reperfusion in rats.

This research concludes that RVX offers strong dose related protection against testicular IR damage. We suggested that the protective effects are mediated, at least partially, by the regulation of the HIF-1α pathway. RVX can exhibit anti-oxidant, anti-inflammatory, and anti-apoptotic actions in testicular tissue that has been treated to IR damage. This is evidenced by lowering the production of HIF-1α and VEGF proteins **(**Fig. 7**)**. These results bring to light the possibility of RVX as a therapeutic drug for the treatment of testicular IR damage. In addition, we highlighted the relevance of HIF-1α as a potential target for further therapeutic interventions in the future. To the best of the authors’ knowledge, this is the first study to investigate the effect of RVX on testicular IR. Moreover, the role of HIF1-α, VEGF, oxidative stress, inflammation, and apoptosis was assessed.

## Electronic supplementary material

Below is the link to the electronic supplementary material.

Supplementary Material 1

## Data Availability

All data generated or analysed during this study are included in this published article [and its supplementary information files].
