# Supplementary material for: Involvement of hypoxia-inducible factor1-alpha in the protective effect of rivaroxaban against testicular ischemia-reperfusion in rats
Source: Sci Rep. 2025 Jul 29;15:27711. doi: 10.1038/s41598-025-10395-2 (PMC12307867; doi:10.1038/s41598-025-10395-2)

Selected represented blot

HIF1 alpha

93 KDA

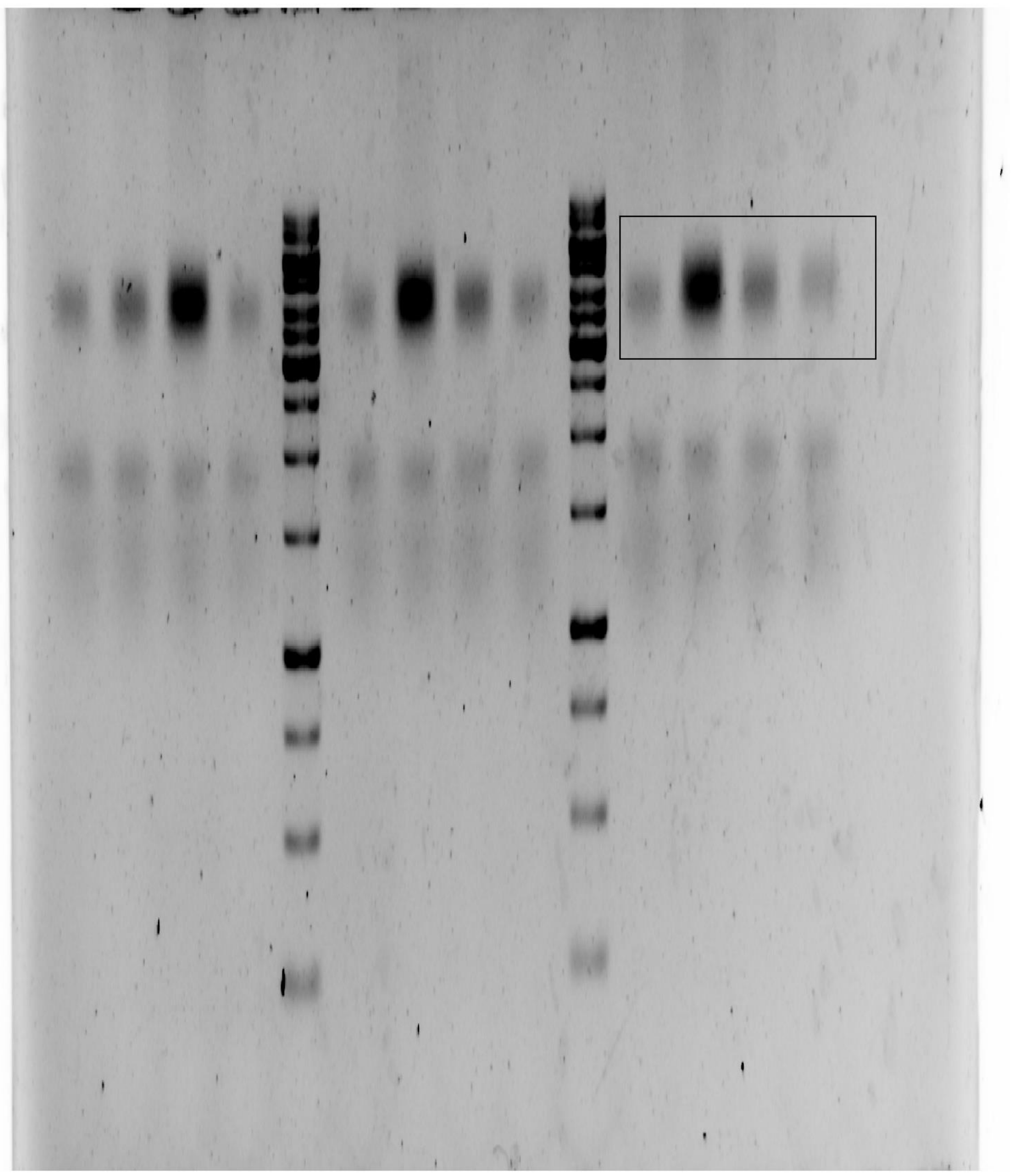

Selected represented blot

VEGF

21 KDA

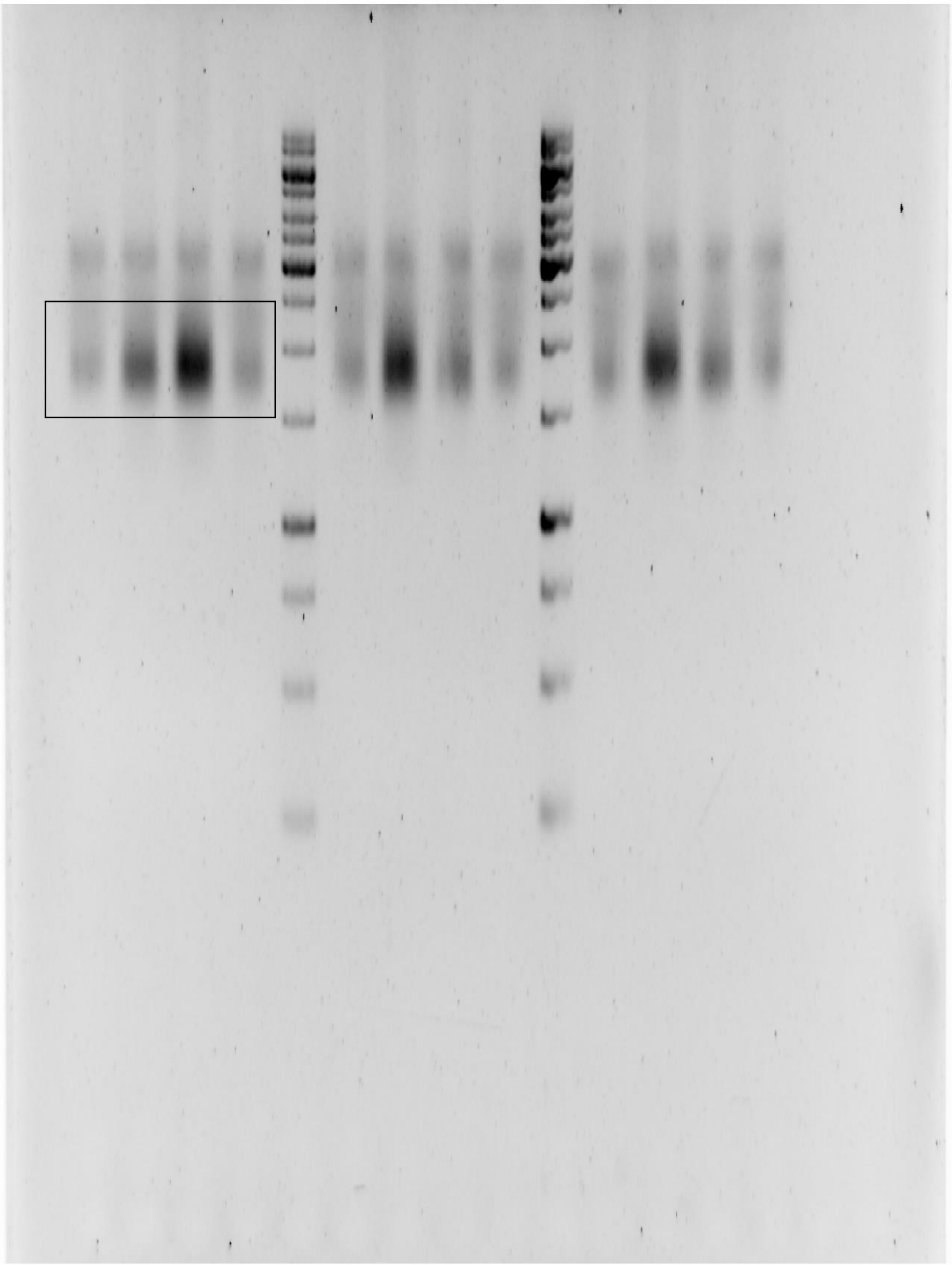

Selected represented blot

B actin

42 KDA

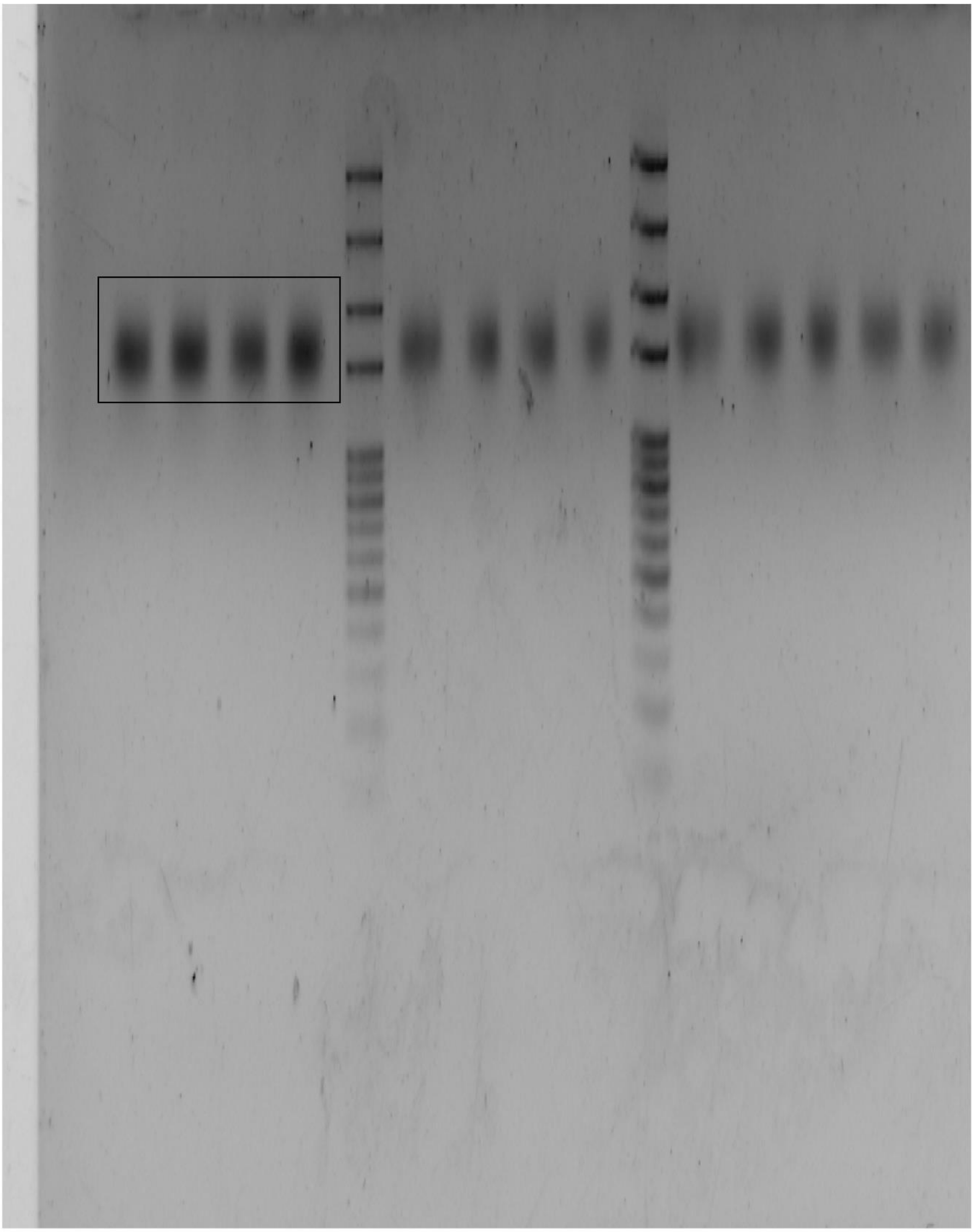

Supplement: Supplementary file 1 — Supplementary Material 1 [file 41598_2025_10395_MOESM1_ESM.pdf]
